# Field-testing phase of the development of individual cognitive stimulation therapy (iCST) for dementia

**DOI:** 10.1186/s12913-016-1499-y

**Published:** 2016-07-08

**Authors:** Lauren A Yates, Vasiliki Orgeta, Phuong Leung, Aimee Spector, Martin Orrell

**Affiliations:** Institute of Mental Health, University of Nottingham, Nottingham, UK; Division of Psychiatry, University College London, London, UK; Research Department of Clinical, Educational and Health Psychology, University College London, London, UK

**Keywords:** Individual cognitive stimulation therapy, Cognitive stimulation therapy, Dementia, Field-testing, Medical research council framework

## Abstract

**Background:**

Cognitive Stimulation Therapy (CST) groups for people with dementia are available nationally, and internationally through voluntary organisations, memory services, and in residential care settings. However, groups may not be accessible or best suited for all. Individual Cognitive Stimulation Therapy (iCST) has been developed to provide another means of accessing CST.

**Methods:**

The programme was field tested by 22 dyads (carers and people with dementia). Dyads were trained in the iCST approach and provided with a manual and accompanying resources. Researchers contacted dyads weekly to provide support and gather adherence data. Quantitative feedback about each session was also collected using ‘Monitoring Progress’ forms. Upon completion of their allocation sessions, researchers interviewed dyads about their experience. In total, nine dyads were followed up. Inductive thematic analysis was performed on the qualitative data. The aims of field testing were to assess the feasibility of the programme, and the appropriateness of the iCST materials.

**Results:**

Sixty-two percent of the themes received an overall ‘high’ rating, and the majority of activities were classed as ‘low’ difficulty. Common barriers to completing sessions were; lack of time, illness, and motivation. Carers felt the manual and resources were ‘good’ and easy to use. Benefits of the programme for the person included; improvements in communication, mood, and alertness. The programme also gave carers insight into the person’s abilities and interests, and provided a new channel of communication. Little support was needed to deliver the programme.

**Conclusions:**

Implementation of the iCST intervention was feasible. However, the majority of dyads completed fewer than three sessions per week. The training and support package appeared to be suitable as carers were able to deliver the intervention without intensive support. Barriers occurred largely as a result of life commitments, rather than problems with the intervention itself. This study was limited by a high loss to follow up rate. The effectiveness and cost effectiveness of iCST were investigated in a large scale randomised controlled trial (RCT).

**Trial registration:**

ISRCTN65945963

Date of trial registration: 05/05/2010

## Background

The use of non-pharmacological interventions in the treatment of dementia is becoming more widespread in the UK. Cognitive Stimulation Therapy (CST) is an evidence-based group programme for people with mild to moderate dementia [1]. CST has consistently been shown to improve the quality of life and cognition of participants [1, 2]. Furthermore the intervention is cost-effective [3]. As a result of its demonstrated evidence base, CST is recommended by several organisations including the National Institute of Clinical Excellence (NICE) [4], National Health Service (NHS) Institute for Innovation and Improvement [5] and Alzheimer’s Disease International (ADI) [6]. Recently, an extended programme of maintenance Cognitive Stimulation Therapy (maintenance CST) was developed [7] and evaluated in a large multi centre randomised controlled trial [8]. Improvements in quality of life were reported for people with dementia at six month follow up, and participating in the programme appears to offer cognitive benefits for those on cholinesterase inhibitors.

CST groups are available nationally, and internationally through voluntary organisations, memory services, and in residential care settings. However, groups may not be accessible or best suited for all. Individuals may be unable to get to local groups if they have health or mobility problems, which make travel difficult, or local services may not offer CST, or have a waiting list for groups. Furthermore, not everyone is comfortable in a group environment, and some individuals have visual or hearing impairments that can make participating in a group challenging [9]. Individual Cognitive Stimulation Therapy (iCST), a one to one home based programme of mentally stimulating activities delivered by a carer has been developed to provide another means of accessing CST. iCST may be used as an alternative, or to compliment group attendance. The programme is comprised of 75 sessions of themed cognitively stimulating activities. Dyads complete up to three, 20–30 min sessions of iCST per week. The recommended duration and frequency of sessions is based on a previous study of home based cognitive stimulation/reality orientation (RO) [10] and constitutes the same ‘dose’ of CST people receive in the group programme (two, 45 min sessions per week) [1]. The iCST package created for the research trial consists of a manual, activity workbook containing paper based resources (eg: images, puzzles) and toolkit of useful items (eg: maps, cards, dominoes).

This is the first study to field test iCST and examine the feasibility of using it in practice, setting the foundation for a randomised controlled trial (RCT). The development of the intervention was extensive, and guided by the Medical Research Council (MRC) framework [11]. The aim of the study was to identify barriers and facilitators to using iCST delivered by the informal carer, to determine the feasibility of the programme in practice including adherence, and to assess the appropriateness of the iCST materials (eg: Manual, Activity Workbook, iCST Toolkit).

## Methods

### Sample

**Fig. 1:**
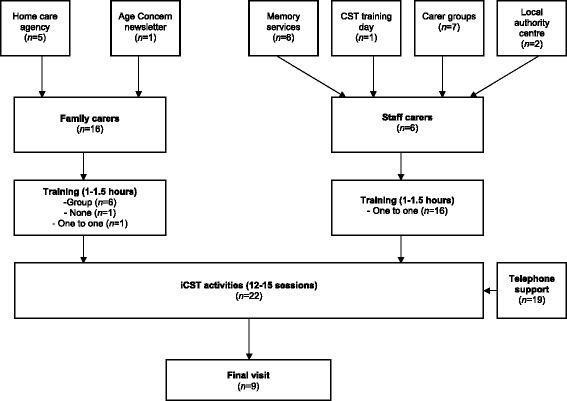
Design of the field-testing phase

Participants were recruited as familial dyads, or pairs of paid carer/client with dementia (see Table 1 for full demographic information). Recruitment of familial dyads took place in North East London boroughs through both voluntary and NHS organisations. Carers and people with dementia were approached at carer support groups or referred by healthcare professionals after memory clinic appointments. The majority of the sample of paid carers was recruited from a private home care agency in North London. One carer approached the research team about participation after seeing an article about the trial in an Age Concern newsletter (see Fig. 1). People with dementia were eligible to participate if they had mild to moderate dementia (meeting the Diagnostic and Statistical Manual of Mental Disorders, 4^th^ Edition [DSM-IV] criteria [12]; & score of 10 or above on the Mini Mental State Examination [MMSE] [13]), were able to communicate and understand communication and provide informed consent, were living in the community and had a carer available to deliver the sessions.

**Table 1:** Demographic information

	Characteristics		Field-testing (%)
People with dementia (*n* = 22)	Gender	Female	11 (50)
	Mean age (years)		81.15 (*SD* = 5.76)
	Ethnicity	White	20 (90)
		Black	1 (5)
		Unknown	1 (5)
Family carers (*n* = 16)	Gender	Female	14 (88)
	Mean age (years)		65 (*SD* = 10.52)
	Ethnicity	White	15 (94)
		Mixed	1 (6)
	Relationship	Spouse	8 (50)
		Child (son/daughter)	8 (50)
	Living status	Spouse living with person	8 (50)
		Adult child living with person	3 (19)
		Person lives alone	5 (31)
	Mean years caring		4.32 (*SD* = 1.87)
Paid carers (*n* = 6)	Gender	Female	5 (83)
	Mean age (years)		42.60 (*SD* = 16.13)
	Living status	Person lives at own home	5 (83)
		Carer lives with person	1 (17)
	Mean years caring		1.75 (*SD* = 1.50)

### Design

The first drafts of the iCST manual and resources were tested, and the data gathered from this phase was used to produce the second drafts of the materials. It is considered best practice to carry out a feasibility study or period of field-testing before investing time, resources and funding in a full study [11]. In interviews and focus groups carried out as part of the development phase [14], carers indicated the value of field-testing, commenting that they would have a clearer idea of the practical issues and the success of the activities if they were able to try the programme.

### Procedure

#### Set up

A standardised training package for carers was created and delivered by the research team (see Fig. 1). Familial dyads were trained in their homes. Although the training was primarily targeted at the carer, in many cases the person with dementia also took an active role in the set up visit, and joined their carer and the researcher for a guided iCST activity. For convenience, a group training session was organized for the agency carers. However, their clients with dementia did not attend. A senior member of the agency was trained, but did not have an eligible client so another carer from the organization was given the iCST materials. The substitute carer did not receive formal training to deliver the intervention. The live-in carer recruited as a result of the Age Concern newsletter was trained on a one to one basis at the person’s home.

At the end of the training session, carers completed a short questionnaire rating their knowledge of iCST, confidence in delivering the programme, perceived level of support required, and training preference (one to one in own home, or group). The data on training preferences was taken to discern which method would be most suitable in the main trial. The researcher recorded general observations about the training, in addition to completing a questionnaire rating the success of the visit, likelihood of the carer engaging with the person with dementia and amount of support anticipated.

#### Field-testing the intervention

The 75-session iCST programme was split between six draft ‘manuals’ and accompanying ‘resource manuals’. Each ‘manual’ served as a ‘how to’ guide for delivering the sessions, and included outlines of the structure and content of each session. The corresponding ‘resource manuals’ contained paper based resources (eg: puzzles, images) for the suggested activities. Participants were allocated between 12 and 15 sessions and advised to complete three, 20–30 min sessions per week (see Fig. 1). Dyads were offered the opportunity to complete an additional selection of sessions once they had completed their original allocation. In order to measure the quality of the materials and adherence to the programme feedback about each activity was captured on ‘Monitoring progress’ forms. The forms required carers to record which sessions they completed and rate aspects of each session including; the person with dementia’s interest, communication, enjoyment, how difficulty they found the session (5-point Likert scale: not at all, a little, moderately, quite a lot, extremely), and their mood (Poor, fair, good, very good, excellent).

#### Support and adherence

Researchers aimed to contact each dyad weekly to obtain qualitative feedback about their experiences and provide advice and support about delivering the programme. The researcher completed a telephone support questionnaire for every contact based on the carer’s responses to the following topics; sessions completed, difficulties, comments about the resources, whether the dyad provided their own resources, enjoyment of the person with dementia, whether any advice was needed about specific issues, and whether the carer had received support with the programme from family or friends. Consent to continue with field-testing was sought at the end of each contact.

#### Final visit

A debrief visit was arranged with dyads who completed all of their allocated sessions (*n* = 9). The researcher interviewed the carer and person about their experience using a questionnaire as a guide. The carer also completed a short questionnaire rating their knowledge, confidence, quality of support received, and perceived level of success in engaging in iCST. Monitoring progress forms were collected at the visit.

### Ethical considerations

Standard procedures were applied in the process of obtaining informed consent from the carers and people with dementia. These included; (a) ensuring dyads were provided with information sheets a minimum of 24 h before providing written consent at the researcher set up visit to allow enough time to consider their participation; (b) offering participants the opportunity to ask questions; and (c) incorporating a clause confirming understanding of information sheets on the consent forms. The right to withdraw participation and any data provided was emphasized by the researcher in the process of obtaining consent. Researchers involved in the study were experienced in the process of obtaining fully informed consent and assessed the capacity of referrals. People with dementia were in the mild to moderate stages, and thus were able to provide informed consent to participate. Familial dyads provided consent at their set up visit. The paid carers, their clients with dementia, and a family member of each nominated client gave written consent prior to the group training session. Family members of Sweet Tree clients were approached for consent as they are consulted about all decisions regarding the care the organisation provides. Ethical approval for this study was obtained through the East London 3 Research Ethics Committee (ref no.10/H0701/71).

### Analyses

Inductive thematic analysis techniques [15] were applied to the written qualitative data obtained from the carer and researcher set up, final visit, and telephone support questionnaires. Categories were derived from the questions on the measures (eg: ‘barriers’) and text pertaining to each category was extracted from the questionnaires. Initially, two researchers analysed the data independently, then the content of the categories was collaboratively reviewed to ensure agreement on category placement.

## Results

Twenty-two dyads took part; 16 of which were family carers, and six of which were paid carers. The mean age of participating family carers was 65 years. The sample of paid carers had a mean age of 43 years. Half of the sample of 22 people with dementia were female. The mean age of participating people with dementia was 81 years (see Table 1).

### Data from set up and final visits

Twenty-one carer set up questionnaires were available for analysis. The set up data from the senior member of staff from the agency with no suitable client was excluded from the final data set, and the carer recruited in their place did not complete a set up questionnaire. In total, 17 researcher questionnaires were completed. Final visit data from both the carer and researcher was obtained for nine dyads. However, of the nine followed up, set up ratings were not available for two dyads. Ratings are shown in Table 2. Post-field-testing ratings show that 57 % (4) carers felt their knowledge of iCST improved, whilst 43 % (3) felt their knowledge remained the same. Seventy-one percent (5) of carers felt just as confident about delivering the intervention at their set up as they did at their final visit, with 43 % (3) noting improvement. Fifty-seven percent (12) of carers preferred a one to one setting for training. All of the set up visits were thought to be highly successful. Low levels of support were anticipated and needed in all cases.

**Table 2:** Set up and final visit ratings derived from carer and researcher measures

	Set up (%)				Final (%)				
Carer	*n* = *21*				*n* = *9*				
	Low	Moderate	High	Missing		Low	Moderate	High	Missing
Knowledge	0	7 (33)	14 (67)	0	Knowledge	1 (11)	0	8 (89)	0
Confidence	0	7 (33)	14 (67)	0	Confidence	0	2 (22)	7 (78)	0
Support needed	18 (86)	1 (5)	1 (5)	1 (5)	Quality of support received	0	0	8 (89)	1 (11)
Researcher	*n* = *17*				Researcher	*n* = *9*			
Success of first session	0	0	17 (100)	0	Success of sessions	1 (11)	1 (11)	7 (78)	0
Ability to engage person in sessions	0	5 (29)	12 (71)	0	Ability to engage person in sessions	1 (11)	2 (22)	6 (67)	0
Anticipated support needed	17 (100)	0	0	0	Amount of support received	9 (100)	0	0	0

Ratings of ‘poor’ , ‘not at all’ and ‘a little’ classified as ‘low’ , ratings of ‘fair’ and ‘quite a bit’ as ‘moderate’ , and ‘good’ , ‘very good’, ‘excellent’ , and ‘a lot’ as ‘high’

Researchers’ final visit ratings of successful engagement were based on the feedback throughout the dyad’s participation, and comments at the visit (Table 2). Sixty-seven percent (6) of dyads were thought to have engaged successfully ‘a lot’ of the time, 22 % (2) ‘quite a bit’ of the time, and one ‘a little’ of the time. The carers (78 %, 7) who felt they had successfully engaged in the programme (‘totally agree’ or ‘agree’) were also considered to have been successful by researchers (‘a lot’ or ‘quite a bit’).

### Monitoring progress data

Complete monitoring progress data was collected for nine dyads. A total of 10 manuals were returned, as one dyad returned two manuals. Within each of the 21 themes, between two and eight sessions were completed. An average of five sessions were completed per theme. On average, three dyads provided feedback about each theme (range = 1–4). The mean number of sessions completed was 12 (Table 3).

**Table 3:** Average number of sessions completed by dyads

Sessions completed (range 4–24)	Number of dyads (%) (*n* = 9)
0–6	1 (11)
7–11	5 (56)
12–16	2 (22)
17–24	1 (11)
Mean number of sessions	11.56 (SD = 5.59)

Scores for the aspects rated on the monitoring progress forms (interest, communication, enjoyment, difficulty and mood) were converted into ‘low’, ‘moderate’ and ‘high’ categories (see Table 4). An overall rating was then generated for each theme. This was a single rating (eg: ‘high’) if there was a majority of one category, or a combined rating (eg: ‘low-moderate) if a majority could not be established. Thirteen of the 21 themes received an overall ‘high’ rating (three or more ‘high’ categories excluding ‘difficulty’). Amongst the remaining themes, four received a ‘Moderate-High’ rating, one a ‘Moderate’ rating, one a ‘Low-Moderate’ rating, and two were categorised as ‘mixed’ because the ratings were split equally between ‘high’ and ‘low’. Seventy-one percent (15) of the themes were placed in the ‘low’ category for difficulty, compared to only 14 % (3) in the ‘high’ category. The remaining three themes were in the ‘low-moderate’ (10 %, 2) or ‘moderate’ categories (5 %, 1). Qualitative comments about each of the themes are also shown in Table 4.

**Table 4:** Quantitative ratings from monitoring progress forms alongside qualitative comments from telephone support questionnaires

Themes	Interest (%)	Communication (%)	Enjoyment (%)	Mood (%)	Overall rating	Positive comments	Negative comments	Difficulty^a^ (%)
Physical Games	H (67)	H (67)	H (67)	H (67)	H	*Good*, *successful session*	*Too heavy*, *cannot be used indoors*, *person does not like skittles*	M/L (67)
Word Association	H (100)	H (67)	H (67)	H (100)	H	*Best session*, *fun*, *easy but gave the person confidence*, *did well in the session*		L (67)
Word Games	H (100)	H (100)	H (100)	H (100)	H	*Good*, *fun*, *gave the person confidence*, *word grid not easy but enjoyable*	*Word search provided looks too difficult*, *jumbled letter grid looks too difficult*	L (75)
Thinking Cards	H (100)	H (100)	H (100)	H (100)	H	*Good fun*, *amusing*	*Too easy*	L (100)
Childhood	H (100)	H (100)	H (100)	H (100)	H	*Interesting images of childhood toys*	*Games shown in images obscure*, *difficult to locate photographs*	M/L (100)
Quiz	H (100)	H (100)	H (100)	H (100)	H	*Fun*, *enjoyed the exercise but didn*’*t do very well*, *did well at music quiz*		L (100)
Faces & Scenes	H (100)	H (100)	H/M (67)	H (83)	H	*Enjoyed looking at images*, *images brought back happy memories*, *stimulated discussion*	*Not as interested in faces as scenes*, *questions for scenes activity difficult*	L (67)
Sound	H (100)	H (100)	M/L (100)	H (100)	H	*Had fun listening to the music*, *lot of discussion generated*, *types of music activity better*, *session went well*	*Difficult due to problems with hearing*, *clips too short*, *too easy*, *too difficult to identify instruments*	H (67)
Number Games	H (50)	H/M (100)	M/L (100)	H (100)	H	*Person did well with dominoes*	*Not interested in dominoes or cards*, *person found the sessions hard*	L (75)
Useful Tips	M (60)	H (80)	H/M (80)	H (83)	H	*Created a lot of discussion*, *session went very well*	*Activity is* ‘*silly*’	L (80)
Art Discussion	H/M (100)	H (100)	H (100)	H (100)	H	*Good*, *lots of discussion*		L (100)
Visual Clips	H (100)	H (100)	M-H (100)	H (100)	H	*Interesting*	*Controversial adverts too difficult*	L (100)
Current Affairs	H (50)	H (50)	M (50)	H (75)	H		*Person had no idea of world events*	M (50)
My Life	M (100)	H (83)	M (67)	H (100)	H/M	*Family tree challenging but enjoyable*, *good questions on game board*, *loved old photos*,	*Not interested in family tree*, *images of occupations need to be clearer*	L (67)
Categorising Objects	M (67)	M (67)	H (67)	H (100)	H/M	*Enjoyed activity and gave lots of reasons and ideas*, *discussion beneficial*, *odd one out cards easy and swift*, *positive session*		L (100)
Household Treasures	M (67)	H (67)	M (100)	H (100)	H/M	*Good*, *happy to identify pairs and discuss images*, *easy but created a lot of discussion*	*Difficult to identify old and new objects*, *topics did not interest the person*	L (100)
Slogans	M (100)	M (100)	L (100)	H (100)	H/M	*Logos enjoyable*	*Logos look too difficult*, *slogans too difficult and too old*	H (100)
Using Money	M/L (67)	M (67)	M (67)	H (67)	M	*Enjoyed talking about currency*, *very good*	*Not interesting*, *no idea of value of money*	L (67)
Orientation	M (71)	M (57)	L (57)	L (57)	M/L	*World map interesting*	*Did not like looking at maps*, *too difficult*, *not interesting or engaging*, *images of landmarks difficult to recognize*	L (57)
Food	L (67)	H (67)	L (67)	H (67)	Mixed	*Images very clear*	*Difficult to recognize some types of food*	H (50)
Being Creative	H (40)	L (60)	H (40)	L (60)	Mixed		*Not interesting*, *person has never done anything creative*	L (100)

Ratings of ‘not at all’ and ‘a little’ classified as ‘low’ and shown abbreviated as ‘L’ , ratings of ‘moderately’ as ‘moderate’ , shown abbreviated as ‘M’ , and ‘quite a bit’ or ‘extremely’ as ‘high’ , abbreviated as ‘H’

^a^For ‘difficulty’ , ‘high’ indicates most difficult

### Data from telephone support questionnaires

The following categories emerged from thematic analysis of the qualitative data gathered (*n* = 19); barriers affecting progress with sessions, difficulties experienced with the programme, feasibility of session structure and duration, iCST manual, iCST resources, perception of sessions and positive outcomes, and support.

### Barriers affecting progress with sessions

Sixty-three percent (12) of carers reported that being busy with ‘life commitments’ affected their progress with the programme. These included job responsibilities, day centre attendance, appointments (eg: hospital visits), holidays, household responsibilities (eg: moving house), and social events (eg: celebrations). Attending to these ‘life commitments’ compromised the amount of time the dyad had available to do sessions together. Forty-two percent (8) of carers found that finding the time to complete sessions was a problem. The experience of health problems was also a common reason for lack of progress with the programme. Issues with the person’s health (32 %, 6) were reported equally as often as issues with the carer’s health (32 %, 6). Another cited barrier to completing sessions was the person’s motivation and willingness to participate (32 %, 6). The barriers described thus far were experienced by both family carers, and paid carers. However, paid carers also reported some events that delayed progress related to their job role, such as taking annual leave.

### Difficulties experienced with the programme

Few difficulties were experienced with the programme itself. However, four carers reported struggling with the orientation discussion at the beginning of each session. Other difficulties mentioned in a small number of cases were; finding delivering the programme ‘hard’, struggling with applying the key principles, and difficulty maintaining conversation. Four carers experienced difficulty engaging the person in the activities.

### Feasibility of session structure and duration

A key concern for carers was ensuring that sessions felt ‘informal’. Some carers adjusted the structure or order of the sessions in an effort to create a more informal atmosphere. Adjustments included breaking up the session into smaller ‘chunks’, completing the orientation and current affairs sections of the session independently from the main activity, or even skipping these completely. Sixteen percent (3) said they were able to complete three sessions per week, whilst the majority of carers were only able to complete one or two. The shortest session duration reported was 20 min, and the longest about an hour.

### iCST Manual

Feedback about the manual was predominantly positive. Carers found the manual easy to use (68 %, 13), describing it as ‘very good’ (58 %, 11), and commenting positively on several aspects of the manual including the layout, size of text, key principles, and ideas provided. Some carers made suggestions for improvements. It was thought that having a selection of ideas for the session warm up would be useful, especially for those who struggled with the orientation discussion.

### iCST Resources

The majority of carers said that they used the resources provided in the activity workbook and toolkit and thought they were ‘good’ (63 %, 12). However, five carers (26 %) supplemented those provided with their own resources. Additional resources included; newspapers, photographs, creative materials (eg: calligraphy kit), puzzle books, board games, and physical games equipment (eg: sponge ball).

### Perception of sessions and positive outcomes

Enjoyment was reported by all field-testing dyads, with the exception of one who refused to engage in the activities. Carers noted that some activities were more enjoyable than others according to the person’s interests (see Table 4). People with dementia were enthusiastic about the activities, showed willingness to participate, and appeared engaged and interested. Carers described positive outcomes for the person such feeling a sense of achievement, being more affectionate, and improvements in the person’s mood, conversation skills and memory. Delivering the programme was also beneficial for carers in many cases. One carer said the activities gave them purpose when spending time with the person and the programme was ‘a lot of help’, whilst another felt they were more tolerant of the person because the programme gave them a greater understanding of how memory works. Some carers reported they were surprised that the person was willing and able to do the activities. Benefits to the relationship between the carer and person were also reported by a carer, who said that the activities brought the pair closer together as it gave them something in common, encouraged them to communicate, which was normally absent, and gave them an opportunity to enjoy themselves and ‘have a laugh’.

### Support

The majority of carers did not seek support from the research team about any issues related to the delivery of the programme. The only support issue raised was by a staff carer, who requested advice about their client’s refusal to engage in the sessions. Eight carers received help in the delivery of the programme from friends, family members (eg: spouses, grand children, siblings) and, in some cases sitters or paid carers.

## Discussion

The purpose of the period of field-testing undertaken during the development phase of the trial was to explore the feasibility of the iCST programme in practice, and gather data about adherence to the programme and the suitability of the intervention materials (iCST Manual, Activity Workbook, iCST Toolkit). The results indicate that with training and support from the research team carers were able to deliver iCST with few difficulties. The main difficulties experienced were not associated with the programme itself, rather finding time and being motivated to do sessions. This was impacted by both expected (eg: moving house) and unexpected events (eg: illness), or commitments (eg: medical appointments). Carers felt the manual and resources were of high quality, easy to use, and visually appealing, and noted benefits of taking part in the programme for both the person and themselves.

### Evaluation of the training and support package

The knowledge and confidence ratings of carers who participated in a debrief visit remained stable or improved in the majority of cases. For those who reported improvement, application of the intervention ‘in practice’ may have served to enhance the understanding of ‘theoretical’ information about the programme provided in the training session [16]. Carers felt the support they had received was of high quality, but rarely requested help beyond the training and researcher initiated calls, which may be indicative that the intervention is easy to deliver, and the training and support package was fit for purpose.

### Appraisal of activities and themes

The majority of the programme themes were highly rated. The least successful themes were ‘orientation’ , ‘food’ , and ‘being creative’ , which received mixed or negative feedback. As a result these themes were subject to review and modification for the second draft of the materials. The relationship between engagement in an activity and assessment of its difficulty was not straightforward. Some participants found it difficult to engage in activities they perceived as ‘easy’. Problems associated with inadequately pitched activities are reported in other studies of activity-based interventions [17, 18]. Adverse effects of activities deemed ‘too easy’, can include boredom, or adoption of repetitive self-stimulating behaviours. At the other end of the scale, if activities are too challenging, the person may be left feeling frustrated, confused or agitated. However, Gigliotti & Jarrott comment that pitching activities at an ‘average’ level may not be the solution, as they may not provide enough stimulation [19]. This makes sense alongside the findings that ‘moderate’ or ‘high’ difficulty ratings did not necessarily predict negative ratings in other dimensions measured (eg: interest, communication, enjoyment). In order to feel stimulated by activities, some individuals may require them to be progressively more challenging, whereas other people take more pleasure in being able to complete tasks with ease. An alternative explanation for the findings may be that people begin to find activities easier if their cognition improves along with participation. Furthermore, the implications of a ‘ceiling effect’ on the potential cognitive benefits of participating in cognitively stimulating activities may need to be considered. If a person is functioning at a high level, the intervention may be of limited use until they reach a certain threshold of impairment in cognitive performance. It is likely that the most effective activities are appropriate for the person’s level of functioning, and this may be subject to change over time. Teri and Logsdon highlight the need for activities to be appropriate for the person’s level of functioning, and acknowledge that although identification of pleasant and appropriately pitched activities can be challenging for carers, and may be related to their creativity, there are benefits to doing so for both the care giver and recipient [17]. For the carer, the benefits of providing appropriate and enjoyable activities for the person include; improved sense of self-efficacy [19], reduction in feelings of burden [19], enhanced relationship with the person [20, 21], and improved wellbeing [22]. For people with dementia, participating in pleasant activities can alleviate depression [23] as well as enhancing the relationship with the carer [21].

In the first draft of the iCST programme materials, two levels of difficulty (level A and level B) were provided for most of the activities, but not all. Since some dyads sometimes struggled to find a balance in the difficulty of the activities, and evidence in the literature emphasises the importance of appropriately tailored activities, activities with one level of difficulty were reviewed, and where appropriate split into two defined levels in the second draft so that carers to increase the choices available. In the main RCT, researchers were available to support carers in tailoring the programme and choosing activities [24].

### Outcomes observed by carers

Although the measures were driven towards obtaining data about dyads’ perceptions of the materials and activities, and experience of the programme, many of the carers commented on the impact taking part in the research had on both themselves, and the person. Carers felt that that participating in the activities was beneficial for the person, and noted improvements in their mood, alertness, and communication during and following the sessions. These outcomes are consistently associated with group CST [2], perhaps indicating that the properties of CST that impact cognition, communication skills, and quality of life are retained in this individualised format. No formal measures of outcomes (eg: cognition, quality of life) were conducted so the positive impacts of participating in the field-testing described by carers can only be treated as anecdotal at this stage. However, the effectiveness of the intervention was not the main focus of the field-testing phase, and has been investigated fully in the main RCT [9, 24].

### Impact on communication

The programme was seen as a catalyst for communication and a source of mutual enjoyment, which encouraged carers to spend time with the person. Communication between the carer and person can become increasingly challenging through the course of dementia. Gillies asserts that this is not simply due to any difficulties with expression and understanding of language the person may develop, but can occur when the nature or boundaries of the relationship between the carer and person change [25]. Both the quantity and quality of conversation can be marred by maladaptive patterns of communication including; the person withdrawing and initiating conversation less, cycles of repetitive questions from the person met with repetitive reminders or frustration from the carer, and getting information wrong or being unable to recall things leading to the carer correcting or ‘testing’ the person. iCST’s focus on opinions rather than facts, emphasis on errorless learning principles, and introduction of new ideas and topics to engage with may serve to alleviate the cycle of these dysfunctional communications, which may account for improvements in communication reported by carers in this study.

Enhancing the quality of dyadic communication can have a profound impact on the person, beyond the pleasure of engaging with, and relating to their carer. According to Kitwood social interactions affect the maintenance of identity [26]. Kitwood’s definition of identity stipulates ‘knowing who one is’ and ‘maintaining a sense of continuity with the past, and some kind of consistency across the course of present life’. Although a person’s sense of identity persists in dementia, cognitive impairment and social-psychological factors (eg: experience of social exclusion, depression) can make maintenance increasingly difficult. As a result, the input of others becomes very important, particularly the way in which they reinforce the person’s ‘life story narrative’ in their behaviour and responses towards them. The person’s carer, as the principal or exclusive source of interaction, will inevitably play a vital role in affirming their ‘narrative’, so poor quality interactions have the potential to exert a deleterious effect on the preservation of self identity. The positive impact of iCST on quality of dyadic communication reported by carers suggests that the programme may have compelling potential wider-reaching benefits for the person related to maintaining identity.

### Benefits of mutual engagement in an activity

The loss of mutual hobbies, leisure activities and social events which sometimes occurs after the onset of dementia can be difficult for carers to come to terms with, and can be a source of stress [27]. The determinants of carer experience of gratification or frustration and burden are complex but, by providing carers and people with a mutual interest, iCST may be used as a simple aid to reduce this stress.

### Benefits of observing person’s skills

Several carers expressed surprise at how ‘well’ the person performed in the activities. Family carers tend to underestimate the person’s ability to perform activities of daily living (ADL) [28], and their perception of the person’s level of impairment often differs to those of independent observers, or professional carers [29]. The closeness of the relationship [29] and carers’ subjective burden [30] are thought to have an influence on these estimations. Observing the person’s success in iCST sessions appeared to develop carers’ understanding of the person’s abilities and interests, and how to cope with the experience of their cognitive impairments.

### Impact of findings on drafting of iCST programme

Feedback from this study, along with data obtained from the interviews and focus groups carried out as part of the development process contributed to the second draft of the intervention materials [31]. Alterations to the first draft of the materials were largely editorial including; correction of spelling and grammar mistakes, improvements to enhance the clarity of instructions, adjustments to the size of some text and images, and changes to the ‘monitoring progress’ forms. No changes were made to the programme in response to feasibility issues identified (eg: finding time for sessions, difficulties with iCST technique) at draft two stage. However, these issues were reviewed as part of the consensus process, resulting in amendments to the guidance provided in the final draft of the manual [31].

### Limitations

A significant limitation of the field-testing study was the small sample size (*n* = 22) and the gaps in both qualitative and quantitative data collected from researchers and dyads. The rate of dyads who did not complete a final visit with a researcher was particularly high (59 %, 13). With only a small number of complete set up and final visit data sets it was difficult to analyze and meaningfully interpret the quantitative ratings provided by carers and researchers. This was also a problem with the data from the monitoring progress forms concerning evaluation of each session theme. Qualitative data was obtained from a bigger proportion of the sample (*n* = 19) (eg: feedback from the telephone support calls, monitoring progress forms, final visit questionnaires) and was used to derive meaning from the ratings.

The iCST themes were rated a varying number of times by a varying number of dyads, therefore less in depth data was obtained for certain themes. The research team aimed to distribute the six manuals as equally as possible, given the numbers recruited, but the type of data gathered was impacted by dropouts and those who did not participate in a final visit. Lack of breadth of data was problematic when identifying activities that needed reworking for draft two of the materials. In some cases, for example, when only two dyads had rated a theme and their feedback was opposing, it was difficult to justify any modifications to the activities.

The sample was not ethically diverse, thus the findings reported may not be representative of the experience or needs of carers and people with dementia of other cultures. As a result, the content of the resources provided for the activities may not fully reflect the interests of a diverse population of participants. However, this may be the case within as well as between cultures. Indeed, as described above, many of the session themes received both positive and negative ratings and some comments were very specific (eg: ‘decided to leave session as mother has never done anything creative’) which suggests that personal preference and interests may ultimately be the most influential factor in the level of enjoyment and engagement in each session theme, as well as how challenging the activities are. A larger and more diverse sample would have been more likely to reveal any stronger trends in appraisals of the themes. However even with a larger sample, the notion of creating an individualised programme of activities ‘suitable for all’ is somewhat paradoxical, so to address this, it is important the intervention is as flexible as possible with the potential to adapt activities to best suit the dyad. Encouragingly, the most successful psychosocial interventions for carers have similar qualities to iCST in that they are tailored to the needs of individuals and involve both the caregiver and recipient.

## Conclusions

The field-testing phase was informative as it demonstrated implementation of the iCST intervention is feasible, and relatively simple. The training and support package appeared to be suitable and effective as carers were able to deliver the intervention without intensive support from the research team. The majority of dyads completed fewer than three sessions per week, with barriers to implementation occurring largely as a result of life commitments and responsibilities, rather than problems with the intervention itself. Despite the small sample size and high rate of loss to follow up, when the data was pooled with data gathered from focus groups and individual interviews [14] sufficient feedback was available to produce the second draft of the materials. The development phase culminated in a two-step consensus process resulting in the final draft of the programme materials. The effectiveness and cost effectiveness of the intervention compared to treatment as usual (TAU) has now been investigated in a large scale RCT.

## Abbreviations

ADI, Alzheimer’s Disease International; ADL, Activities of Daily Living; APA, American Psychiatric Association; CST, Cognitive Stimulation Therapy; DSM-IV, Diagnostic and Statistical Manual of Mental Disorders (4th Edition); HTA, Health Technology Assessment; iCST, Individual Cognitive Stimulation Therapy; LSE, London School of Economics; Maintenance CST, Maintenance Cognitive Stimulation Therapy; MMSE, Mini-Mental State Examination; MRC, Medical Research Council; NHS, National Health Service; NICE, National Institute of Clinical Excellence; NIHR, National Institute for Health Research; RCT, Randomised Controlled Trial; TAU, Treatment as usual; UCL, University College London.

## References

[1] Spector A, Thorgrimsen L, Woods B, Royan L, Davies S, Butterworth M, Orrell M (2003). A randomised control trial investigating the effectiveness of an evidence-based cognitive stimulation therapy programme for people with dementia. Br J Psychiatry.

[2] Woods B, Aguirre E, Spector AE, Orrell M (2012). Cognitive stimulation to improve cognitive functioning in people with dementia. Cochrane Database Syst Rev.

[3] Knapp M, Thorgrimsen L, Patel A, Spector A, Hallam A, Woods B, Orrell M (2006). Cognitive stimulation therapy for people with dementia: cost-effectiveness analysis. British J Psychiatry.

[4] National Institute of Health and Clinical Excellence (NICE) (2006). Supporting people with Dementia and their Carers in Health and Social care.

[5] NHS Institute for Innovation and Improvement (2011). An economic evaluation of alternatives to antipsychotic drugs for individuals living with dementia.

[6] Prince M, Bryce R, Ferri C: *World Alzheimer Report: The benefits of early diagnosis and intervention.* Alzheimer’s Disease International; 2011. [http://www.alz.co.uk/research/WorldAlzheimerReport2011.pdf].

[7] Aguirre E, Spector A, Hoe J (2011). Development of an evidence based extended programme of Maintenance Cognitive Stimulation Therapy (CST) for people with dementia. Nonpharmacol Ther Dement.

[8] Orrell M, Aguirre E, Spector A, Hoare Z, Woods RT, Streater A, Donovan H, Hoe J, Knapp M, Whitaker C, Russell I (2014). Maintenance cognitive stimulation therapy for dementia: single-blind, multicentre, pragmatic randomised controlled trial. Br J Psychiatry.

[9] Orrell M, Yates LA, Burns A, Russell I, Woods RT, Hoare Z, Moniz-Cook E, Henderson C, Knapp M, Spector A, Orgeta V (2012). Individual Cognitive Stimulation Therapy for dementia (iCST): study protocol for a randomized controlled trial. Trials.

[10] Onder G, Zanetti O, Giacobini E (2005). Reality orientation therapy combined with cholinesterase inhibitors in Alzheimer’s disease: randomised controlled trial. Br J Psychiatry.

[11] Craig P, Dieppe P, Macintyre S, Michie S, Nazareth I, Petticrew M (2008). Developing and evaluating complex interventions: the new Medical Research Council guidance. BMJ.

[12] American Psychiatric Association (1994). Diagnostic and Statistical Manual of Mental Disorders.

[13] Folstein MF, Robins LN, Helzer JE (1983). The Mini-Mental State Examination. Arch Gen Psychiatry.

[14] Yates LA, Orrell M, Spector A, Orgeta V (2015). Service users’ involvement in the development of individual Cognitive Stimulation Therapy (iCST) for dementia: A qualitative study. BMC Geriatr.

[15] Thomas DR (2006). A general inductive approach for analyzing qualitative evaluation data. Am Journal Eval.

[16] Van de Ven AH, Johnson PE (2006). Knowledge for theory and practice. Acad Manage Rev.

[17] Teri L, Logsdon RG (1991). Identifying pleasant activities for Alzheimer’s disease patients: The Pleasant Events Schedule-AD. Gerontologist.

[18] Gigliotti CM, Jarrott SE (2005). Effects of horticulture therapy on engagement and affect. Can J Aging.

[19] Gitlin LN, Winter L, Burke J, Chernett N, Dennis MP, Hauck WW (2008). Tailored Activities to Manage Neuropsychiatric Behaviours in Persons with Dementia and Reduce Caregiver Burden: A Randomized Pilot Study. Am J Geriatr Psychiatry.

[20] Hellstrom I, Nolan M, Lundh U (2005). ‘We do things together’. A case study of couplehood in dementia. Dementia.

[21] Hellstrom I, Nolan M, Lundh U (2007). Sustaining couplehood. ‘Spouses’ strategies for living positively with dementia. Dementia.

[22] Teri L, Logsdon RG, Uomoto J, McCurry SM (1997). Behavioural treatment of depression in dementia patients: a controlled clinical trial. J Gerontol B Sci Soc Sci.

[23] Marshall MJ, Hutchinson SA (2001). A critique of research on the use of activities with person with Alzheimer’s disease: A systematic literature review. J Adv Nurs.

[24] Orgeta V, Leung P, Yates L, Kang S, Hoare Z, Henderson C, et al. Individual cognitive stimulation therapy for dementia: a clinical effectiveness and cost-effectiveness pragmatic, multicentre, randomised controlled trial. Health Technol Assess. 2015;19(64):1-108. 10.3310/hta19640 PMC4781454 26292178

[25] Gillies B. Continuity and loss: The carer’s journey through dementia. Dementia. 2011;11:657–76.

[26] Kitwood T. Dementia reconsidered: The person comes first. Buckingham: Oxford University Press; 1997.

[27] Pinquart M, Sorensen S. Associations of stressors and uplifts of caregiving with caregiver’s burden and depressed mood: a meta-analysis. J Gerontol (B Psy Sci Soc Sci). 2003;58(2):112–28. 10.1093/geronb/58.2.p112 12646594

[28] Zank S, Frank S. Family and professional caregivers’ ratings of dementia symptoms and activities of daily living of day care patients: do differences change over time? Aging Ment Health. 2002;6(2):161–5. 10.1080/13607860220126790 12028885

[29] Moye J, Robiner WM, Mackenzie TB. Depression in Alzheimer patients: discrepancies between patient and caregiver reports. Alzheimer Dis Assoc Disord. 1993;7(4):187–201. 10.1097/00002093-199307040-00001 8305187

[30] Mangone CA, Sanguinetti RM, Baumann PD, Gonzales RC, Pereyra S, Bozzola FG, Gorelick PB, Sica REP. Influence of feelings of burden on the caregivers’ perception of the patient’s functional status. Dementia. 1993;4:287–93. 10.1159/000107335 8261026

[31] Yates LA, Leung P, Orgeta V, Spector A, Orrell M. The development of individual Cognitive Stimulation Therapy (iCST) for dementia. Clinical Interv Aging. 2015;10:95–104. 10.2147/CIA.S73844 PMC4283984 25565792

